# Functional anatomy of entheses and enthesis organs: A celebration of Professor Mike Benjamin's contribution to enthesis biology

**DOI:** 10.1111/joa.70043

**Published:** 2025-10-05

**Authors:** Hannah Shaw

**Affiliations:** ^1^ School of Biosciences Cardiff University Cardiff UK

**Keywords:** entheses, enthesis, enthesis organ, osteotendinous juction

## Abstract

This review celebrates the work of Professor Mike Benjamin, whose anatomical research transformed our understanding of entheses ‐ the sites where tendons, ligaments and other connective tissues attach to bone. This review aims to provide an overview of Professor Benjamin's foundational concepts, including the enthesis organ, functional entheses and the synovio‐entheseal complex and their relevance to musculoskeletal health and disease. Entheses are biomechanically complex regions that accommodate the transition between compliant soft connective tissues and rigid bone by natural macroscopic and microscopic adaptations that reduce stress concentration. Macroscopically, tendons and ligaments often flare near their attachment sites, increasing surface area. Microscopically, entheses are classified as fibrous or fibrocartilaginous, with the latter displaying a zonal organisation that includes uncalcified and calcified fibrocartilage. These zones provide a graded transition in stiffness, reducing the risk of tissue failure and enables gradual bending of collagen fibres. Mechanical loading is essential for the normal development of the enthesis and is required to maintain its biomechanical properties in the adult. The enthesis organ concept, one of Professor Benjamin's most significant contributions, recognises that entheses are rarely isolated structures. Instead, they are part of a functional unit comprising adjacent tissues including sesamoid and periosteal fibrocartilages, bursae, fat pads and retinaculae which collectively dissipate mechanical stress. Adipose tissue and synovium at these sites may also play immunological and proprioceptive roles, and its involvement in neurovascular invasion has implications for pain and pathology. However, beyond direct tendon‐bone attachments, functional entheses describe regions where tendons and ligaments interact with bone at a distance from the insertion but share structural and functional characteristics with classical entheses. The development of these concepts highlights Professor Benjamin's integrative approach to research and will continue to underpin research in musculoskeletal biology, pathology and tissue engineering, as well as inspire generations of anatomists.

## Graphical abstract

This review celebrates the work of Professor Mike Benjamin, whose anatomical research transformed our understanding of entheses. Entheses are biomechanically complex regions that accommodate the transition between soft connective tissues and bone by natural macroscopic and microscopic adaptations that reduce stress concentration.

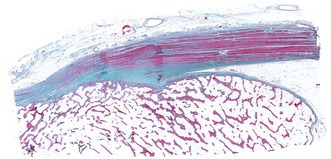

## 1 INTRODUCTION

This reviewand special issue is dedicated to Professor Mike Benjamin. Mike's research career was largely dedicated to investigating the structure and function of connective tissues and their attachments to bone (also known as ‘entheses’). Mike's contributions to this field of biology raised the profile of their functional and clinical importance. His observations of the macroscopic and microscopic anatomy of connective tissues led to the development of several important concepts (Table 1), and this work continues to stimulate research in the field today. However, as detailed in his obituary (published in the Journal of Anatomy by Moxham (2018)), Mike was also an excellent and committed teacher. He supported the training and development of many anatomists working in education and research today, leaving a significant and ongoing legacy.

**TABLE 1:** An overview of the enthesis related concepts developed by Professor Mike Benjamin and colleagues during his career.

Concept	Definition
Enthesis organ	A collection of tissues adjacent to the enthesis which help to reduce stress concentration and demonstrate degenerative changes.
Functional enthesis	Sites where the characteristic features of an enthesis organ (i.e. periosteal and sesamoid fibrocartilages) are present but there may not be any close or direct attachment of these to bone.
Synovio‐entheseal concept	The close structural and functional relationship of synovium and entheses.

This review provides an overview of some of Mike's seminal work on the anatomy of entheses as a prelude to the papers in this special issue of the Journal of Anatomy from the Anatomical Society Symposium delivered at the 116th Annual Meeting of the Anatomische Gesellschaft in Berlin (September 2022) called ‘Connective Tissue: Form, Function and Fabrication’. This paper was written in consultation with Dr. Jim Ralphs, Mike's long‐term friend, cycling companion and collaborator who was intimately involved in the work on structure, composition and development of the enthesis.

## 2 ENTHESES

The point at which a tendon, ligament, joint capsule or dense fibrous connective tissue attaches to bone is known as an enthesis. Entheses are the intersection between soft and hard tissue, and as a consequence, they are a potential region of stress concentration and failure (Benjamin et al., 1986). Interestingly, natural adaptations at entheses make them highly resistant to rupture, but they are vulnerable to overuse injuries (known as enthesopathies) for example, insertional Achilles tendinopathies, tennis and golfers' elbow (Milz et al., 2004). Additionally, entheses are the primary target for a group of rheumatic diseases known collectively as seronegative spondyloarthropathies (Benjamin & McGonagle, 2001). Entheses also need to be properly reconstituted following the surgical re‐attachment/replacement of tendons or ligaments to bone (Gögele et al., 2023), and the accurate development of artificial biomaterials to assist repair of the interface is an ongoing challenge (Paxton et al., 2010). An understanding of the normal anatomy of the enthesis is therefore fundamental to advancing these areas of research.

### 2.1 Why are entheses a potential site of stress concentration?

The main reason why entheses are a region of stress concentration is because it is a meeting point between two tissues with very different physical properties ‐ a relatively soft, compliant dense fibrous connective tissue (Young's modulus of the human gastrocnemius tendon in vivo is ~1.2 GPa (Maganaris & Paul, 1999; Maganaris & Paul, 2002)); and a stiff, hard bone (Young's modulus of compact bone is 15–25 GPa (Currey, 1984)). However, adding to the stress concentration at these sites is the change in ‘insertional angle’ which takes place during movement, as well as the ground reaction forces that they experience, which both increase the risk of damage at the tissue interface (Benjamin et al., 2002, 2006).

### 2.2 How do entheses reduce stress concentration?

Entheses demonstrate a number of basic adaptations at macroscopic and microscopic levels which function primarily to dissipate stress:

#### 2.2.1 Macroscopic adaptations

Gross anatomical observations of tendons and ligaments show that they typically flare out near their attachment sites. This simple adaptation increases the surface area for anchorage and therefore decreases the stress concentration per unit area (Benjamin et al., 2006; Schlecht, 2012), as demonstrated by the trapezoidal insertion of flexor digitorum profundus (FDP) (Mortimer et al., 2021). Through the investigation of 29 different entheses in the body, Benjamin, Redman, et al. (2004) hypothesized that endotenon fat may act as a packing and lubricating material between bundles of collagen fibres where this enthesis flaring takes place. However, Rossetti et al. (2017) have more recently demonstrated, through the scanning and electron microscopy of porcine entheses, that broadening of the attachment zone may also be created by the ‘unravelling’ of the tendon collagen fibres into thinner fibres at the tissue interface.

It has also been observed that tendons/ligaments rarely attach to a single isolated location in an attempt to dissipate stress. Many will attach to neighbouring structures (e.g. bone, tendon, or ligament) via fibrous connections (Shaw & Benjamin, 2007). There are several specific examples of this interconnection theory, including the fibres of the quadriceps tendon becoming continuous with the patellar ligament over the anterior surface of the patellar (Benjamin et al., 2008), and some fibres from the Achilles tendon (or its paratenon) are continuous with the plantar fascia over the posterior surface of the calcaneus (Singh et al., 2021; Stecco et al., 2013) or anchor into the spiral fat pad in the sole of the foot (Shaw et al., 2008). This has been debated in the literature, however, with Snow et al. (1995) considering some of these continuations being lost in the adult.

In a further attempt to reduce stress concentration, tendons and ligaments may combine to form a common insertion point for example, the lateral collateral ligament of the elbow and the tendon of the extensor muscles of the forearm (Milz et al., 2004). This idea of a functionally interrelated network of connective tissues in the body relates to the concept of the ectoskeleton, which Mike discussed in his paper on the importance of fascia in the limbs and back (Benjamin, 2009). In a correlational idea, retinacula are frequently associated with tendons, especially where there is a likelihood for insertional angle change at the enthesis. This is most obvious in the digits, but another good example is the retinacula of the Achilles tendon, which is formed by the inferior part of the crural fascia (Shaw & Benjamin, 2007). The presence of this retinaculum reduces the insertional angle change but also prevents kinking of the tendon, which may result in a potential weak point within the tendon. Interestingly, around 9% of all ballet injuries involve the Achilles tendon, and the dancers wear strapping as supplementary ‘retinacula’ to help support the action of the crural fascia (Benjamin, 2009).

#### 2.2.2 Microscopic adaptations

**FIGURE 1:**
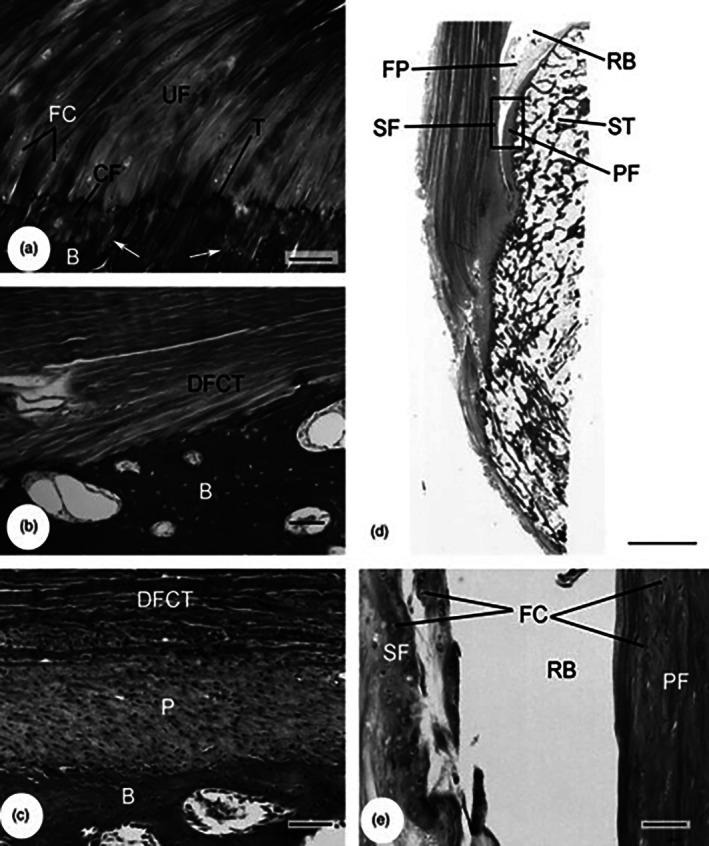
Histological structure of fibrous and fibrocartilaginous entheses (a) The fibrocartilaginous enthesis of the human Achilles tendon and its zonal arrangement of calcified (CF) and uncalcified (UF) fibrocartilage (FC). A calcification front, the tidemark (T) separates hard from soft tissue, which is distinct from the boundary between tendon and bone (b) which is marked by an irregular cement line (arrows). (b) The bony fibrous enthesis of the human pronator teres. (c) The periosteal fibrous attachment of an equine ligament. The ligament (DFCT) attaches indirectly to the bone via the thick periosteum (P). The enthesis organ concept: (d) Low‐power view of the enthesis organ associated with the human Achilles tendon. The enthesis organ comprises the enthesis (e), sesamoid (SF) and periosteal (PF) fibrocartilages, the retrocalcaneal bursa (RB) and the tip of Kager's fat pad (FP). ST, superior tuberosity. (e) Higher‐power view of the sesamoid and periosteal fibrocartilages representative of the region enclosed by a rectangle in (d). (Shaw & Benjamin, 2007).

The adaptations for reducing stress concentration at entheses extend to the microscopic level. Entheses may be categorized into two main types: fibrous and fibrocartilaginous, depending on the histological character of the tissue interface (Figure 1).

Fibrous entheses are usually present where tendons/ligaments attach to the diaphysis of a long bone and can be further sub‐classified into ‘bony’ or ‘periosteal’ depending on whether the tendon inserts indirectly into the periosteum or directly into the bone (Benjamin et al., 1986, 2002). Either way, it is proposed that collagen fibres from the tendon or ligament (often known as the perforating fibres of Sharpey) insert into the bone to facilitate anchorage (Hems & Tillmann, 2000). It is thought that the initial attachment via the periosteum allows for migration of the tendon as the bone lengthens to ensure the relative position of the tendon or ligament remains unchanged during growth (as reviewed by Benjamin et al. (2002)).

**FIGURE 2:**
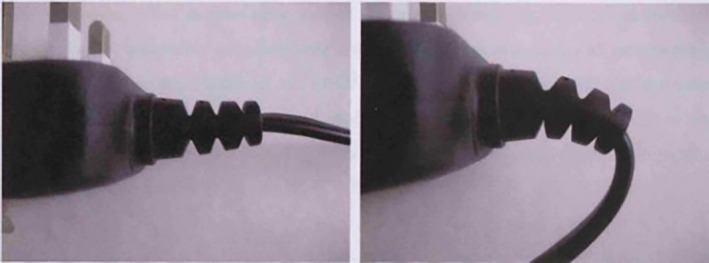
The fibrocartilage at the enthesis is analogous to the grommet on an electrical plug, facilitating gradual bending of the cord (tendon) to prevent wear and tear (Shaw, 2007).

By contrast, fibrocartilaginous entheses possess a fibrocartilaginous plug between the tendon/ligament and the bone (Benjamin et al., 1986). This fibrocartilage acts as an intermediate material, stiffening the tendon at the insertion site, therefore ensuring a gradual transition between the compliant tendon and hard bone. This variation in biomechanical properties along the length of the insertion site has been quantitatively evidenced (Genin et al., 2009; Thomopoulos et al., 2003). The fibrocartilage also promotes the gradual bending of the collagen fibres as they approach the bone to ensure that fibre bending is not focused intensely at the tissue interface, therefore reducing the risk of damage (Benjamin et al., 2002). The fibrocartilage is therefore analogous to the grommet on the plug which promotes bending and prevents fraying of the cable (Figure 2).

Interestingly, the amount of fibre bending differs both within a single attachment site and in different anatomical regions of the attachment (Peniche Silva et al., 2022). For example, Achilles tendon fibres insert at right angles to the bone (Milz et al., 2002), while those of the FDP insert at approximately 30° (Mortimer et al., 2021). However, there has been limited recognition of such anatomical variations in the research literature and the functional or clinical implications that they may have (e.g. increased risk of avulsion at some sites).

A typical fibrocartilaginous enthesis will consist of four zones: *dense fibrous connective tissue* of the tendon or ligament, *uncalcified fibrocartilage* (UCFC), a relatively thin layer of *calcified fibrocartilage* (CFC) and the underlying *bone*. Interestingly, the border between the uncalcified and calcified fibrocartilage, known as the tidemark, is relatively straight/smooth, while the actual tendon‐bone junction (between the CFC and bone) is very complex with interlocking, jigsaw‐like projections, as illustrated by Milz et al. (2002). This interlocking arrangement ensures that the tendon‐bone interface increases surface area to facilitate anchorage, while the smooth tidemark reduces the risk of damage caused by shearing movement of the tendon. Immunohistochemical analysis has demonstrated that the UCFC and CFC of the enthesis have a similar composition to that of articular cartilage, except for the abundance of type I collagen. Both contain type II collagen and the large aggregating proteoglycan, aggrecan which functionally allow the tissue they are contained within to resist compression. The fibrocartilage therefore functions as a stretching break, effectively preventing the tendon from narrowing at the insertion when load is applied, as well as allowing a gradual bending of the collagen fibres (Benjamin et al., 1986; Milz et al., 2002).

Several authors have demonstrated that the greater the degree of bending that occurs at an insertion site (i.e. the greater the insertional angle change) the more fibrocartilage is present within the enthesis (Benjamin & Ralphs, 1998; Evans et al., 1990). It is therefore not surprising that immunohistochemical analysis of the presence of aggrecan between trained and detrained rodents showed that it was responsive to mechanical loading (Frizziero et al., 2011) and highlighted the essential role of loading in rehabilitation from injury. However, increased fibrocartilage may not always be considered a good thing, with Ferretti et al. (1983) illustrating increased fibrocartilage and ossification in patients with Jumper's knee (either patellar or quadriceps enthesopathy).

The seminal work by Thomopoulos and colleagues has also emphasized the essential role mechanical loading plays in the normal development of the enthesis. Using botulinum toxin A to paralyse supraspinatus muscle in mice, Thomopoulos et al. (2007) showed that up until 14 days of postnatal development, the enthesis developed normally. However, after 14 days, muscle paralysis had a detrimental impact on the deposition of aggrecan and bone mineralization at the enthesis. Schwartz et al. (2013) further demonstrated that this reduction in loading had a quantitative impact and diminished the mechanical properties of the enthesis.

It is important to recognize however, that stress dissipation also occurs on the bone side of the entheses. Contrary to what most may expect, there is a distinct absence of thick compact bone at entheses; instead, trabecular bone is commonly observed adjacent to the tendon interface (Toumi et al., 2006). Functionally, this absence of compact bone allows the tendon to slightly deform the associated bone, again reducing a sharp contrast in stiffness at the enthesis and helping to reduce the risk of failure. The arrangement of the trabeculae near the enthesis is modified to dissipate mechanical load well into the bone, which also can be seen radiographically (Canoso, 1998). Unsurprisingly, therefore, the loading created by the tendon has an impact on the arrangement of the trabeculae themselves. Toumi et al. (2006) have provided evidence that supports this hypothesis, indicating that the orientation of the trabeculae strongly reflects the direction of tendon pull. Consequently, we should not think of the enthesis as ending at the bony interface, but should include a diffuse, difficult‐to‐define region of bone beneath the surface as well. This important inclusion of a region of bone deep to the entheses may also go some way in explaining why inflammation of the bone (osteitis) and enthesopathy are frequently linked and observed together clinically (Benjamin & McGonagle, 2009).

Due to the adaptations present, the enthesis is often considered to be the toughest part of the tendon‐bone unit, with avulsion of the bone itself the most commonly observed consequence of acute loading (Golman et al., 2021; Rossetti et al., 2017). A particularly common form of avulsion injury observed is ‘Sever's disease,’ which results in the separation of the Achilles tendon along with the posterior part of the calcaneus at its growth plate—effectively, the entire enthesis (with its associated bone) is pulled away by contraction of the muscle (Benjamin et al., 2002).

## 3 ENTHESIS ORGANS

One of Mike's most significant advancements in the field was the ‘discovery’ of the enthesis organ, that is, the recognition that entheses are usually associated with adjacent tissues which also help to reduce the stress concentration at the tendon‐bone interface (Benjamin & McGonagle, 2001; Benjamin, Moriggl, et al., 2004). This theory was proposed following a histological review of signs of degeneration at numerous entheses and the observation that degeneration is seen at the enthesis and other tissues near it. Therefore, to understand the enthesis and its disorders, you must understand that the enthesis itself and its adjacent tissues form a functional unit—this unit was termed the *‘enthesis organ’* and can be defined as a group of related tissues serving to reduce stress concentration (Benjamin, Moriggl, et al., 2004). Mike's research highlighted that enthesis organs are widespread throughout the body, but the easiest one to understand is that of the Achilles tendon, which was also called the ‘Premiere’ enthesis by Canoso (1998).

The Achilles tendon enthesis organ can most easily be understood by considering what happens during plantar and dorsiflexion of the foot at the ankle joint. To provide an increased moment arm and greater mechanical advantage, the Achilles tendon attaches to the inferior 2/3rds of the calcaneus and during dorsiflexion, the tendon presses against the superior tuberosity of the calcaneus (Figure 1). This distributes some of the load from the tendon that would otherwise fall on the enthesis itself onto the adjacent bone. As the tendon presses against the bone in dorsiflexion, the tendon adapts and fibrocartilage develops within it to resist compression. This extension of the enthesis fibrocartilage is known as the ‘*sesamoid fibrocartilage*’ because it is within the tendon (i.e. analogous to a sesamoid bone). In addition, the bone against which the tendon presses also becomes fibrocartilaginous; this is called the *‘periosteal fibrocartilage’* (Canoso, 1998; Milz et al., 2002). In order to reduce the friction between the two fibrocartilages, a synovial bursa (in this example, the retrocalcaneal bursa) is present. Finally, protruding into the bursa is the tip of a fat pad in this case called ‘Kager's fat pad’. The fat pad acts like a variable plunger moving in and out of the bursa to prevent pressure changes in the bursa as the insertional angle changes, preventing the formation of adhesions and spreading synovial fluid between the fibrocartilage (Theobald et al., 2006). It has also been considered to act as a reverse retinaculum, filling the gap between the tendon and bone, thereby preventing any kinking and subsequent weakness to the tendon. Collectively, all these structures, together with the enthesis itself, constitute the ‘enthesis organ.’ It has been well recognized in the literature that there are obvious parallels between the Achilles tendon enthesis organ and a synovial joint, i.e. two articular cartilages separated by a synovial cavity into which a fat pad protrudes. Indeed, (Bywaters, 1965), referred to the Achilles enthesis as being half joint, half bursa. He also coined the term ‘Bywater's sign,’ which refers to the radiological observation when Kager's fat pad fails to protrude into the retrocalcaneal bursa due to its inflammation. Interestingly, in some patients, the superior tuberosity of the calcaneus is enlarged, known as Haglund's deformity; the enlarged size of the tuberosity can predispose the patient to retrocalcaneal bursitis due to increased friction in this region. Hagland's deformity is commonly surgically treated by resecting the enlarged tuberosity. However, given the important role that the sesamoid and periosteal fibrocartilages play in dissipating stress at the enthesis, the efficacy of this treatment must be questioned as it may in time lead to further problems in this region.

## 4 FUNCTIONAL ENTHESES

By analysing a variety of different enthesis organs, it was also observed that tendons and ligaments commonly insert into troughs or pits, meaning that the bone adjacent to the insertion can function as a miniature bony pulley and, like the superior calcaneal tuberosity, provide a greater mechanical advantage for the tendon to act (Benjamin, Moriggl, et al., 2004). The same is true for tendons and ligaments that insert close to the joint space. This is classically observed at the talar attachment of the anterior talofibular ligament (ATFL). The ATFL enthesis is effectively an *intra‐articular* enthesis organ, as it has a sesamoid fibrocartilage, which opposes the articular cartilage of the joint, which is functionally analogous to the periosteal fibrocartilage separated by a bursal space. These observations lead to the idea that other regions of tendons and ligaments may have functions and characteristics similar to those of the enthesis, even without the direct attachment of the tendon or ligament to the bone. These regions are known as *‘functional entheses’* and are most obvious in regions where tendons and ligaments pass around bony pulleys such as the medial or lateral malleolus (Benjamin & McGonagle, 2001; Kumai et al., 2002).

## 5 ADIPOSE TISSUE AT ENTHESES

Adipose tissue has been identified as an integral part of the enthesis organ, but it has been proposed that the adipose tissue may play a number of additional roles. It was observed that Kager's fat pad contains macrophages and lymphocytes, which were also observed on the surface of the associated sesamoid fibrocartilage. It was therefore proposed that the fat pad may function as an immune organ at healthy entheses, similar to the role of the greater omentum in the abdomen, providing a location for lymphocytes and macrophages to reside, remove any debris generated by wear and tear, and possibly protecting the enthesis from infection (Shaw et al., 2007).

Additionally, nerve fibres and proprioceptive nerve endings have been observed in the adipose tissue at the entheses. It may therefore be hypothesized that the fat pad functions as a giant mechanoreceptor and provides proprioceptive information during foot movement (Benjamin, Redman, et al., 2004; Shaw et al., 2007). The presence of nociceptive nerve fibres indicates that the fat pad may also be a source of pain in pathologies, particularly when it becomes compressed in retrocalcaneal bursitis. Furthermore, it has been observed that the fat pad can be the primary source of neurovascular invasion into the typically avascular and aneural enthesis fibrocartilage (Alfredson & Ohberg, 2005; Kristoffersen et al., 2005; Ohberg & Alfredson, 2002). It is hypothesized that wear and tear to the enthesis may lead to a loss of sulphated proteoglycans at the enthesis (as shown through reduced toluidine blue staining) which may allow the in‐growth of neurovascular bundles (Shaw et al., 2007). The concentration‐dependent inhibition of nerve and endothelial growth by aggrecan has been demonstrated in vitro and there are clear parallels with neurovascular invasion in the intervertebral disc (Johnson et al., 2005).

## 6 SYNOVIO‐ENTHESEAL CONCEPT

Finally, the adipose tissue at the entheses is usually lined with synovium and leads us to another concept outlined during Mike's career, called the synovio‐entheseal concept (SEC) (McGonagle et al., 2007). This relates to the close integration and functional interdependence of synovium and entheses. These are two very different tissues located in close proximity to each other—one highly vascular and intrinsically pro‐inflammatory (the synovium) and a normally avascular and anti‐inflammatory tissue (the enthesis). Under normal circumstances, the synovium is probably beneficial to the enthesis—lubricating and nourishing associated fibrocartilages. However, when there is mechanical damage to some part of the enthesis organ, this could potentially trigger an inflammatory reaction in the synovium (Benjamin & McGonagle, 2009) and demonstrated by the co‐existence of degenerative changes at entheses and inflammatory changes in the synovium (McGonagle et al., 2007).

## 7 CONCLUSION

Mike was a dedicated member of the Anatomical Society and a pioneer in the field of connective tissue biology with over 150 publications, many of which were in the Journal of Anatomy. He played a central role in the development of our modern appreciation of the functional anatomy of tendons, ligaments and entheses, coining the terms ‘enthesis organ’, ‘functional entheses’ and ‘synovio‐enthesial concept’. He highlighted the fundamental importance of observing the gross and histological structure of the body, and these observations formed the foundation of his research. Mike's work will continue to be the anatomical foundation for research in the field of connective tissue biology, pathology, and tissue engineering/reconstruction.

## Data Availability

Research data are not shared.
